# Supplementary material for: Semisupervised Contrastive Learning for Bioactivity Prediction Using Cell Painting Image Data
Source: J Chem Inf Model. 2025 Jan 6;65(2):528–43. doi: 10.1021/acs.jcim.4c00835 (PMC11776044; doi:10.1021/acs.jcim.4c00835)
Supplement: Supplementary file 1 — ci4c00835_si_001.pdf [file ci4c00835_si_001.pdf]

Semi-supervised Contrastive Learning for Bioactivity Prediction using Cell Painting Image Data

*David Bushiri Pwesombo[1,2], Carsten Beese[1], Christopher Schmied[1,3], Han Sun[1,2]**

[1]Research Unit Structural Chemistry and Computational Biophysics, Leibniz-Forschungsinstitut für Molekulare Pharmakologie, Berlin 13125, Germany

[2]Technische Universität Berlin, Institute of Chemistry, 10623, Berlin, Germany

[3]EU-OPENSCREEN, Berlin 13125, Germany

*Email: hsun@fmp-berlin.de

Table S1. Total number of annotations and number of unique annotations for MeSH class and repurposing hub annotations.

Annotations	BBBC022	BBBC036
Compounds with MeSH classes	839	1079
Unique MeSH classes	298	317
Unique single-label MeSH classes	221	253
Compounds with Drug Repurposing Hub MoA annotations	773	1534
Unique Drug Repurposing Hub MoA classes	291	592
Compounds with Drug Repurposing Hub target annotations	587	720
Unique Drug Repurposing Hub target classes	659	774

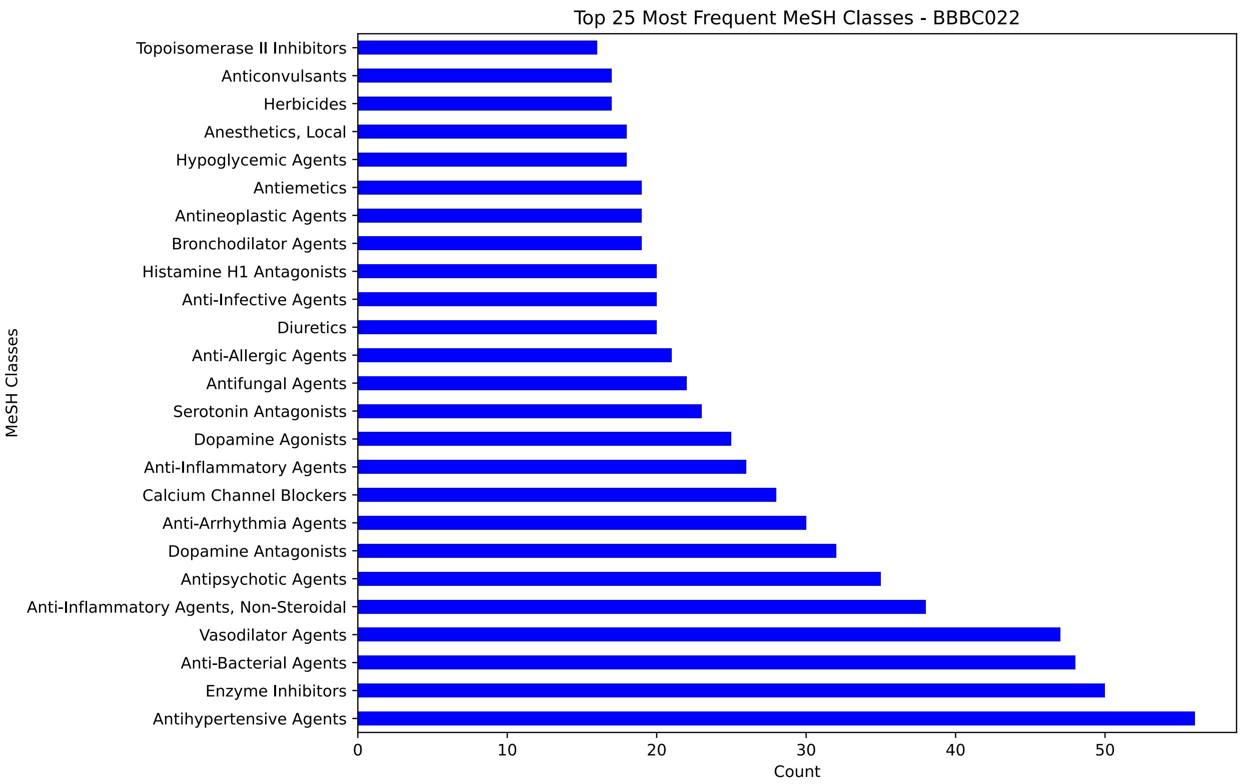

Figure S1. Histogram of the 25 most frequent MeSH classes in the BBBC022 dataset. The distribution provides insight into the prevalence of all available MeSH classes.

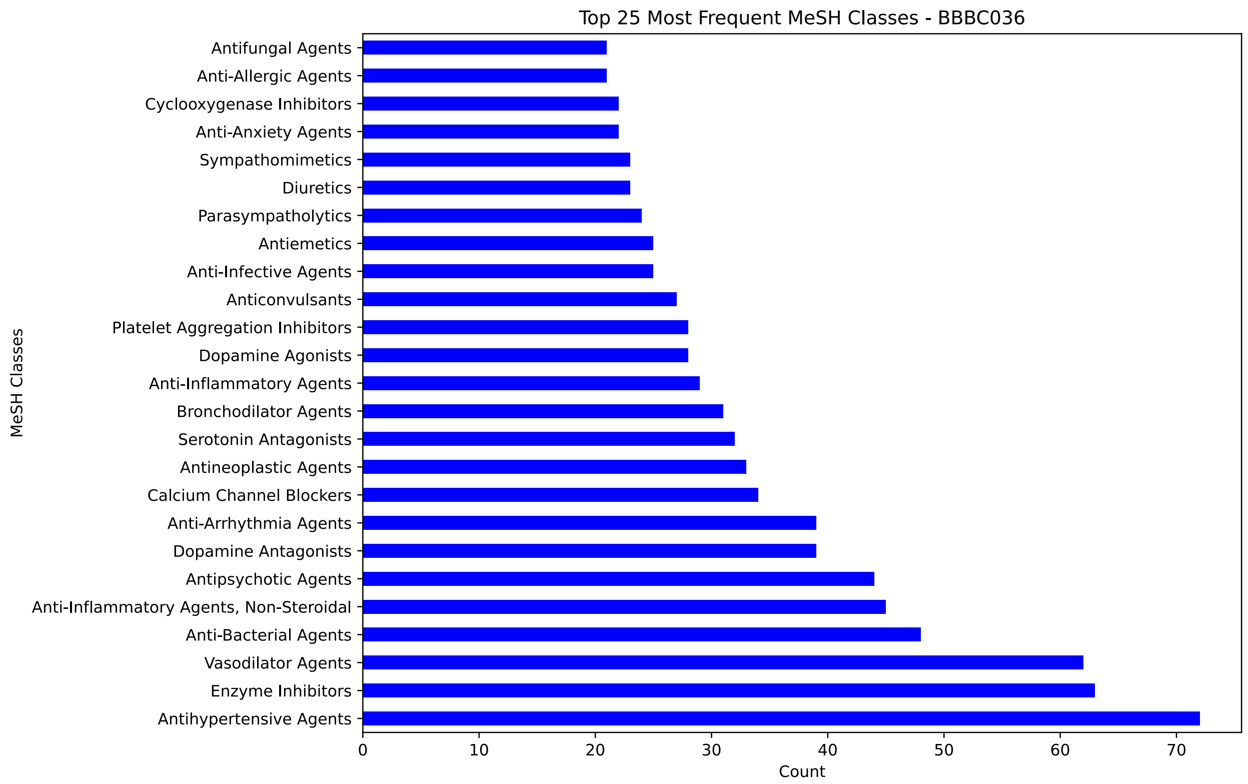

Figure S2. Histogram of the 25 most frequent MeSH classes in the BBBC036 dataset. The distribution provides insight into the prevalence of all available MeSH classes.

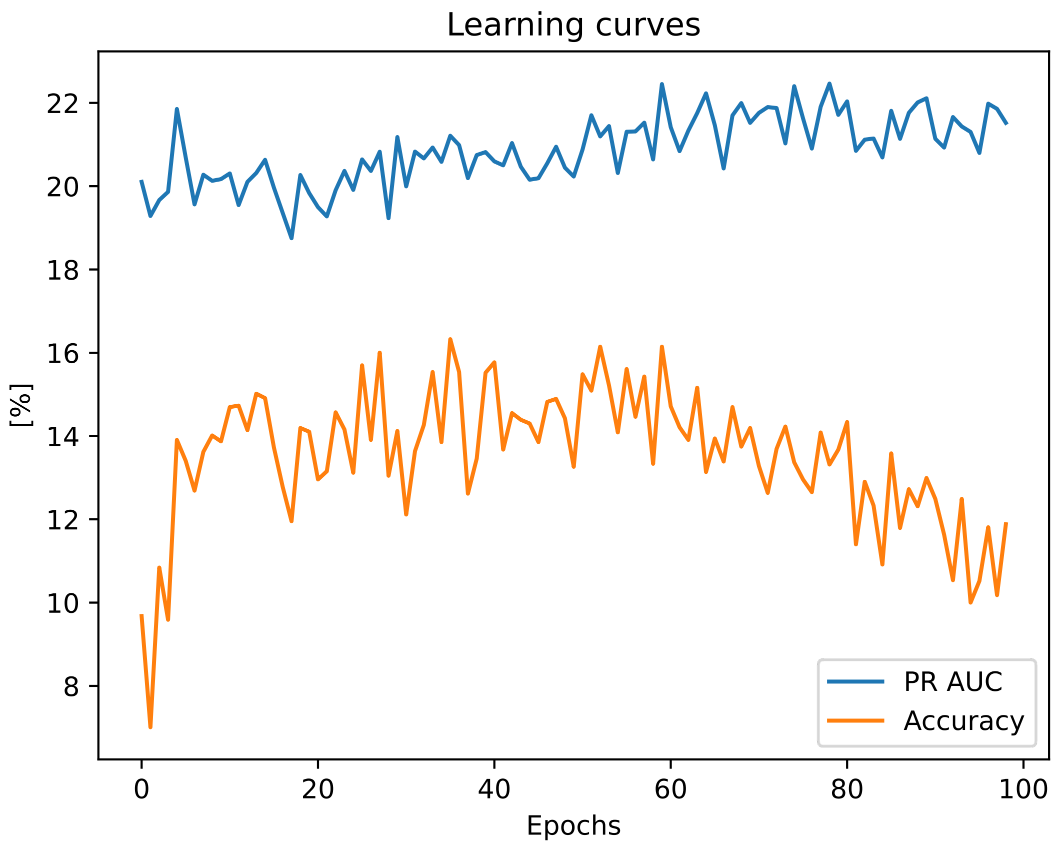

Figure S3. Exemplary learning curves of an MLP using SemiSupCon features from the BBBC022 dataset for Drug Repurposing Hub MoA prediction, with multi-label accuracy and PR AUC as evaluation metrics.

Table S2. An inductive SemiSupCon approach was compared against baselines on downstream classification on the BBBC022 dataset. CellProfiler, transductive Con, transductive DINO and a fully supervised trained ResNet50 were used as baselines. Downstream MLP was evaluated for multi-label prediction of MeSH classes and Drug Repurposing Hub annotations for all methods except for the supervised trained ResNet50. The supervised ResNet50 was only trained and evaluated for multi-label MeSH class classification (5-CV used). 80 % of the compounds were in the training set and the remaining 20 % were in the test set.

Strategy	MeSH class accuracy [%]	Drug Repurposing Hub MoA accuracy [%]	Drug Repurposing Hub targets accuracy [%]
CellProfiler	0	0	0
DINO-tiff	0	0	0
Con	0.39	0.59	**0.64**
SemiSupCon-inductive	**0.64**	**0.86**	0
Supervised ResNet50	0.00 ± 0.00	**-**	-

Table S3. Predictive performance for 11 MeSH classes with highest F1-score based on the single-label RF trained on DINO(BBBC022) features. Only classes with F1-score above 10 % shown. Mean precision for all MeSH classes was 0.74 %.

**MeSH class**	**Precision [%]**	**Recall [%]**	**F1-score [%]**
Herbicides	13.59	23.73	13.41
Cardiotonic Agents	13.52	15.83	13.22
Dopamine Uptake Inhibitors	17.23	11.94	10.87
Enzyme Inhibitors	9.00	10.44	8.94
Antipsychotic Agents	7.10	9.91	8.16
Anti-Inflammatory Agents	7.22	9.35	7.61
Anti-Bacterial Agents	4.52	9.84	6.01

MeSH class	Precision [%]	Recall [%]	F1-score [%]
Antinematodal Agents	6.32	6.11	5.63
Anti-Inflammatory Agents, Non-Steroidal	3.25	4.55	3.71
Insecticides	3.69	4.31	3.41
Dopamine Agonists	3.29	3.45	3.11

Table S4. Predictive performance for 16 MeSH classes with highest F1-score based on the single-label RF trained on Con(BBBC022) features. Only classes with F1-score above 10 % shown. Mean precision for all MeSH classes was 1.05 %.

MeSH class	Precision [%]	Recall [%]	F1-score [%]
Antineoplastic Agents, Phytogenic	28.27	37.22	32.09
Anti-Inflammatory Agents	24.36	29.17	24.84
Antinematodal Agents	20.67	22.22	16.90
Insecticides	13.88	11.67	12.29
Antidepressive Agents	15.77	11.67	11.31
Antipsychotic Agents	10.01	11.20	10.20
Dopamine Agonists	12.75	11.03	9.45
Herbicides	10.69	8.96	8.95
Dopamine Antagonists	6.95	15.83	8.58

MeSH class	Precision [%]	Recall [%]	F1-score [%]
Anti-Bacterial Agents	7.37	9.98	8.14
Enzyme Inhibitors	8.39	11.60	7.89
Adrenergic beta-Antagonists	17.62	6.67	7.58
Hypoglycemic Agents	4.81	15.93	6.39
Serotonin Antagonists	6.60	6.62	6.10
Cardiotonic Agents	8.29	4.44	5.65
Antihypertensive Agents	2.93	3.32	3.07

Table S5. Predictive performance for 15 MeSH classes with highest F1-score based on the single-label RF trained on CellProfiler(BBBC022) features. Only classes with F1-score above 10 % shown. Mean precision for all MeSH classes was 1.07 %.

MeSH class	Precision [%]	Recall [%]	F1-score [%]
Cardiotonic Agents	40.83	31.90	33.74
Antineoplastic Agents, Phytogenic	23.54	30.56	25.79
Antinematodal Agents	25.35	18.89	21.12
Herbicides	17.28	23.67	18.45
Anti-Inflammatory Agents	13.38	34.26	17.55
Enzyme Inhibitors	12.30	17.37	13.08

**MeSH class**	**Precision [%]**	**Recall [%]**	**F1-score [%]**
Antipsychotic Agents	8.85	20.16	11.98
Anti-Bacterial Agents	5.33	34.24	9.15
Anti-Inflammatory Agents, Non-Steroidal	3.81	11.77	5.55
Insecticides	5.15	5.00	5.01
Antihypertensive Agents	4.71	6.97	4.73
Vasodilator Agents	5.86	5.71	4.52
Serotonin Antagonists	4.85	3.70	3.75
Dopamine Agonists	4.15	3.98	3.39
Anti-Infective Agents	2.79	4.49	3.04

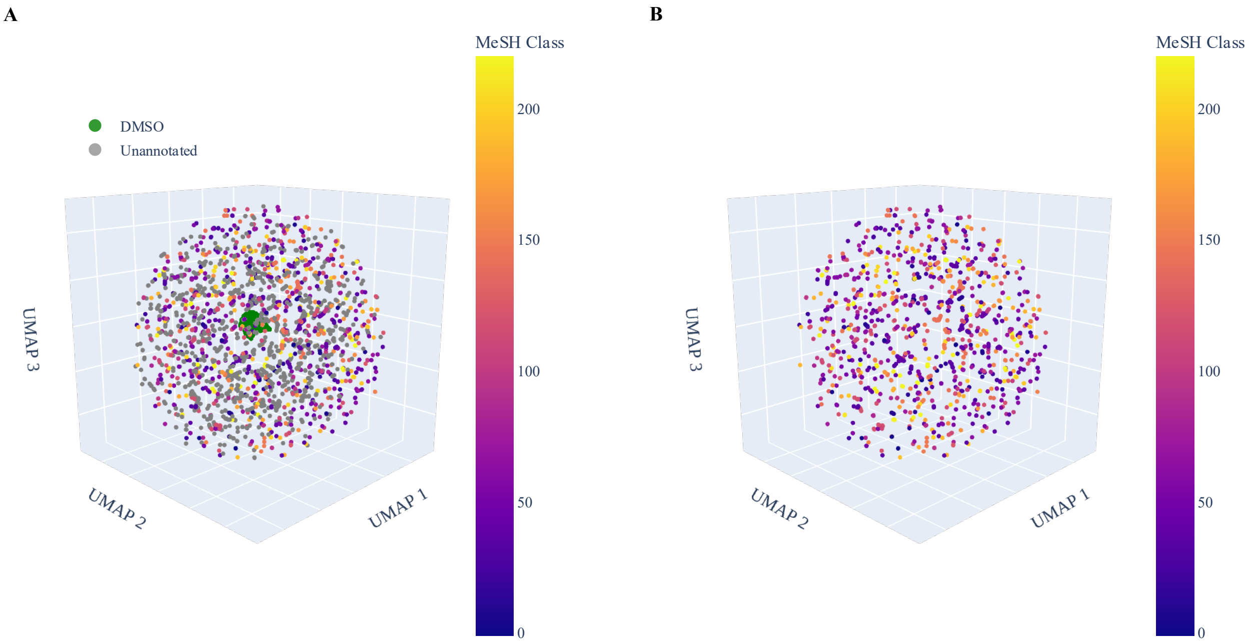

Figure S4. 3D visualization of the phenotypical space from the SemiSupCon(BBBC022) model with UMAP. Data points were colored according to their MeSH classes (compounds with multiple MeSH classes were assigned to their first MeSH class). Grey data points indicate unavailability of a MeSH class, while green data points represent the DMSO controls. (A) Visualization with all data points. (B) Visualization with only the annotated subset.

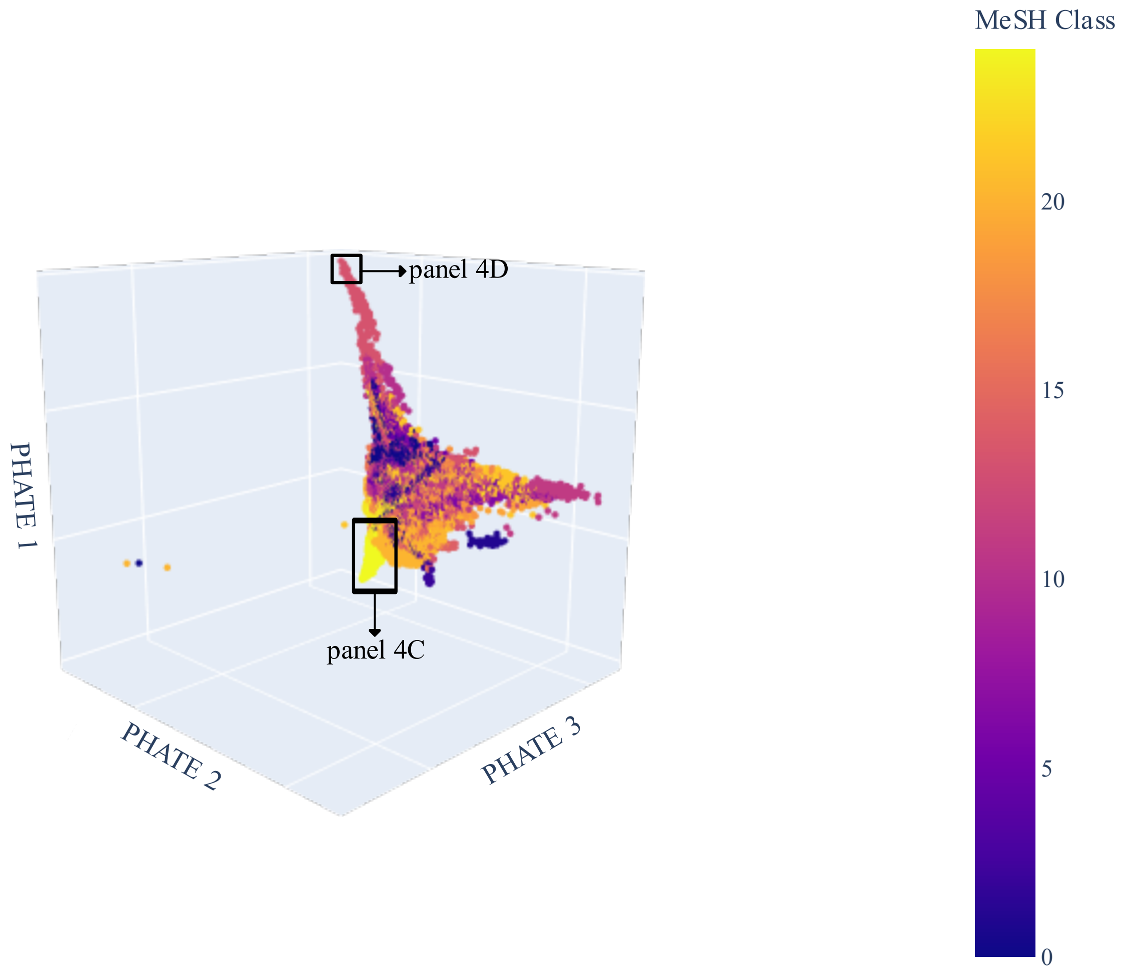

Figure S5. A 3D visualization of the phenotypic space derived from SemiSupCon (BBBC022) features using the PHATE algorithm, showing only the subset of data points belonging to the 25 MeSH classes with the highest F1-scores. Color codes are sorted according to the F1-scores, from highest to lowest.

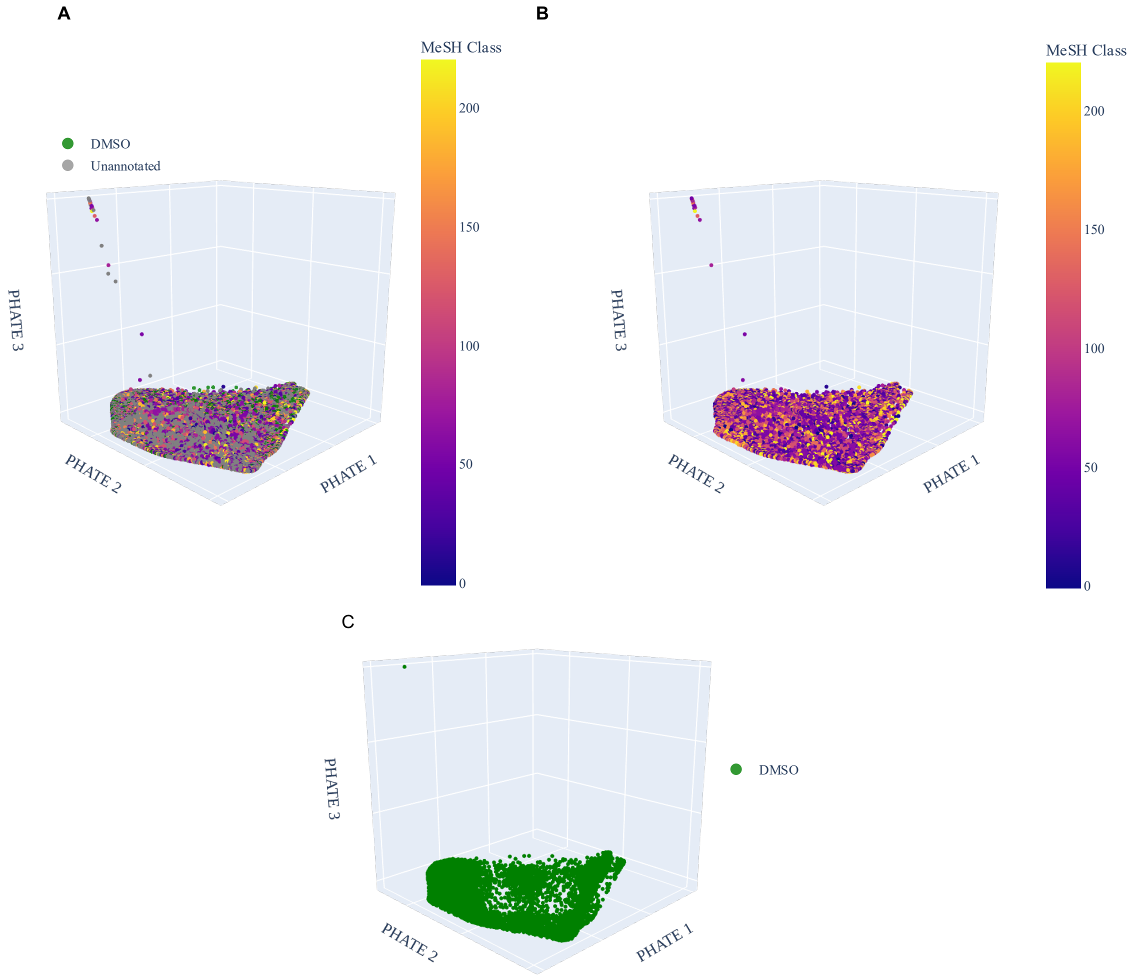

Figure S6. PHATE visualization of the CellProfiler profiles calculated from the BBBC022 dataset. Data points are colored according to their MeSH classes (compounds with multiple MeSH classes were assigned to their first MeSH class). Grey data points indicate unavailability of a MeSH class, while green data points represent the DMSO controls. (A) Visualization with all data points. (B) Visualization with only the annotated subset. (C) Visualization with only the DMSO control.

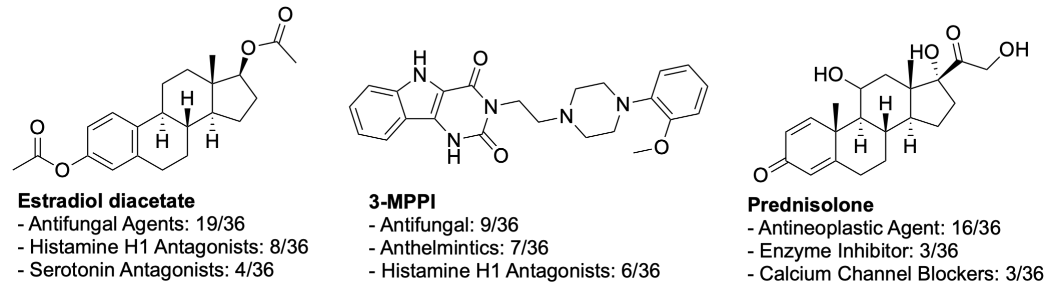

Figure S7. Compounds from top 10 unannotated compounds in the BBBC022 dataset with the lowest contrastive loss, where predictions of MoAs could not be validated through a literature search using a single-label RF model. Predictions were made for each replicate of a compound (36 in total), and for each compound, the three MeSH classes with the highest number of predictions are shown.

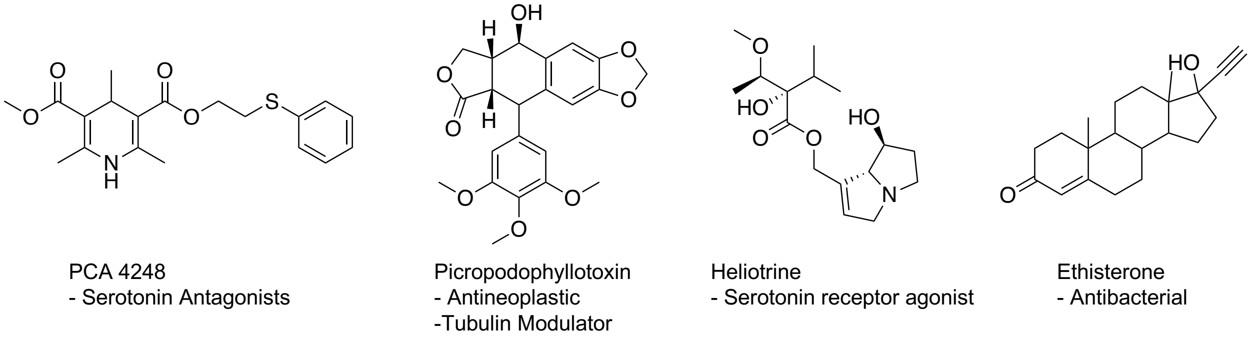

Figure S8. Four predictions of MoAs, confirmed through literature search, for the 10 unannotated compounds with the lowest contrastive loss in the BBBC022 dataset using a multi-label RF model trained on SemiSupCon(BBBC022) representations. PCA 4248 was identified to be a serotonin antagonist[1]. Picropodophyllotoxin was confirmed to be both a tubulin modulator and an antineoplastic compound[2]. Heliotrine was confirmed to be a serotonin receptor agonist[3] and Ethisterone was confirmed to have antibacterial activity[4].

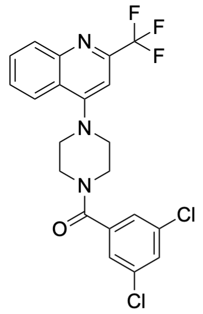

BRD-K00732328
- Antipsychotic

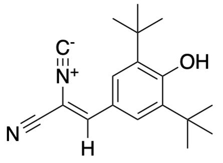

BRD-K38477985
- Ionophore

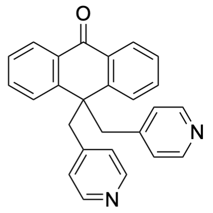

BRD-K42748308
- Antipsychotic

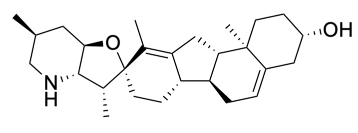

Cyclopamine
- Dopamine antagonist

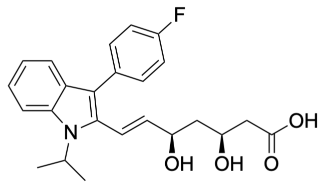

Fluvastatin
- HMGCR inhibitor

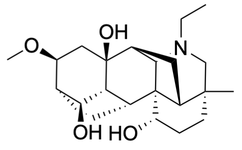

Karakoline
- Antihypertensive

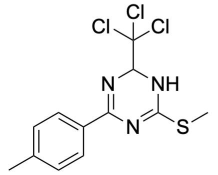

BRD-A31835455
- Tubulin modulator

Figure S9. Seven MoA predictions that were confirmed through literature searches from top 25 profiles of unannotated compounds in the BBBC036 dataset with the lowest contrastive loss. The predictions were generated using a multi-label RF model trained on SemiSupCon(BBBC036) features. Specifically, BRD-K00732328 was confirmed as an antipsychotic compound[5], BRD-K38477985 as an Ionophore[6], BRD-K42748308 as an antipsychotic compound[7], Cyclopamine as a dopamine antagonist (Cyclopamine inhibits the hedgehog pathway)[8], Fluvastatin as a HMGCR inhibitor[9]. Evidence suggests Karakoline functions as an antihypertensive compound[10], and tubulin as one of the targets of BRD-A31835455 in ChEMBL[11].

References

(1) Martins, M. A.; Lima, M. C. R.; Bozza, P. T.; Castro Faria Neto, H. C.; Silva, P. M. R.; Sunkel, C. E.; Cordeiro, R. S. B. Intererence of the PAF Receptor Antagonist, PCA 4248, with the Rat Pleurisy Evoked by Inflammatory Mediators or Allergen. *Eur. J. Pharmacol.* **1993**, *237* (1), 17–22. https://doi.org/10.1016/0014-2999(93)90087-X.

(2) Linder, S.; Shoshan, M. C.; Gupta, R. S. Picropodophyllotoxin or Podophyllotoxin Does Not Induce Cell Death via Insulin-like Growth Factor-I Receptor. *Cancer Res.* **2007**, *67* (6), 2899. https://doi.org/10.1158/0008-5472.CAN-06-0635.

(3) Schmeller, T.; El Sahzly, A.; Wink, M. Allelochemical Activities of Pyrrolizidine Alkaloids: Interactions with Neuroreceptors and Acetylcholine Related Enzymes. *J. Chem. Ecol.* **1997**, *23*, 399–416. https://doi.org/10.1023/B:JOEC.0000006367.51215.88.

(4) Chopra, S.; Matsuyama, K.; Hutson, C.; Madrid, P. Identification of Antimicrobial Activity among FDA-Approved Drugs for Combating Mycobacterium Abscessus and Mycobacterium Chelonae. *J. Antimicrob. Chemother.* **2011**, *66* (7), 1533–1536. https://doi.org/10.1093/jac/dkr154.

(5) Kazantsev, A. G. Compositions and Methods for Modulating Sirtuin Activity. US2009259044A1, October 15, 2009.

(6) Terada, H.; Fukui, Y.; Shinohara, Y.; Ju-ichi, M. Unique Action of a Modified Weakly Acidic Uncoupler without an Acidic Group, Methylated SF 6847, as an Inhibitor of Oxidative Phosphorylation with No Uncoupling Activity: Possible Identity of Uncoupler Binding Protein. *Biochim. Biophys. Acta BBA - Bioenerg.* **1988**, *933* (1), 193–199. https://doi.org/10.1016/0005-2728(88)90070-9.

(7) Chorvat, R. J.; Zaczek, R.; Brown, B. S. Ion Channel Modulators That Enhance Acetylcholine Release: Potential Therapies for Alzheimer's Disease. *Expert Opin. Investig. Drugs* **1998**, *7* (4), 499–518. https://doi.org/10.1517/13543784.7.4.499.

(8) Parga, J. A.; Rodriguez-Pallares, J.; Blanco, V.; Guerra, M. J.; Labandeira-Garcia, J. L. Different Effects of Anti-Sonic Hedgehog Antibodies and the Hedgehog Pathway Inhibitor Cyclopamine on Generation of Dopaminergic Neurons from Neurospheres of Mesencephalic Precursors. *Dev. Dyn. Off. Publ. Am. Assoc. Anat.* **2008**, *237* (4), 909–917. https://doi.org/10.1002/dvdy.21481.

(9) Zhang, T.; Bai, R.; Wang, Q.; Wang, K.; Li, X.; Liu, K.; Ryu, J.; Wang, T.; Chang, X.; Ma, W.; Bode, A. M.; Xia, Q.; Song, Y.; Dong, Z. Fluvastatin Inhibits HMG-CoA Reductase and Prevents Non-Small Cell Lung Carcinogenesis. *Cancer Prev. Res. Phila. Pa* **2019**, *12* (12), 837–848. https://doi.org/10.1158/1940-6207.CAPR-19-0211.

(10) Xu, X.; Xie, X.; Zhang, H.; Wang, P.; Li, G.; Chen, J.; Chen, G.; Cao, X.; Xiong, L.; Peng, F.; Peng, C. Water-Soluble Alkaloids Extracted from Aconiti Radix Lateralis Praeparata Protect against Chronic Heart Failure in Rats via a Calcium Signaling Pathway. *Biomed. Pharmacother.* **2021**, *135*, 111184. https://doi.org/10.1016/j.biopha.2020.111184.

(11) *Compound Report Card*. https://www.ebi.ac.uk/chembl/compound_report_card/CHEMBL1485537/ (accessed 2024-03-08).
